# A true denial or a false confession? Assessing veracity of suspects' statements using MASAM and SVA

**DOI:** 10.1371/journal.pone.0198211

**Published:** 2018-06-01

**Authors:** Bartosz Wojciech Wojciechowski, Minna Gräns, Moa Lidén

**Affiliations:** 1 Institute of Psychology, University of Silesia, Katowice, Poland; 2 Faculty of Law, Uppsala University, Uppsala, Sweden; Xuanwu Hospital, Capital Medical Universty, CHINA

## Abstract

Previous research on statement analysis has mainly concerned accounts by witnesses and plaintiffs. In our studies we examined true and false statements as told by offenders. It was hypothesized that SVA and MASAM techniques would enhance the ability to discriminate between true and false offenders' statements. Truthful and deceptive statements (confessions and denials) were collected from Swedish and Polish criminal case files. In Experiment 1, Swedish law students (N = 39) were asked to assess the veracity of statements either after training in and usage of MASAM or without any training and using their own judgements. In Experiment 2, Polish psychology students (N = 34) assessed veracity after training in and usage of either MASAM or SVA or without prior training using their own judgements. The veracity assessments of participants who used MASAM and SVA were significantly more correct than the assessments of participants that used their own judgements. Results show, that trained coders are much better at distinguishing between truths and lies than lay evaluators. There were significant difference between total scores of truthful and false statements for both total SVA and MASAM and it can be concluded that both veracity assessment techniques are useful in assessing veracity. It was also found, that the content criteria most strongly associated with correct assessments were: logical structure, contextual embedding, self—depreciation, volume of statement, contextual setting and descriptions of relations. The results are discussed in relation to statement analysis of offenders' accounts.

## Introduction

Enhancing legal actors ability to make correct suspects' statements veracity judgments is of pivotal importance both during criminal investigations and court proceedings. However, previous studies show that overall accuracy of professional criminal investigators’, prosecutors’, judges’ as well as ordinary people’s veracity judgments do not exceed 55%, when non specialized deception detection tool is used [1,2,3] (.Scientists have therefore engaged in developing special techniques to enhance legal actors’ accuracy of veracity assessments. Thus, being able to create a reliable method for this purpose would not only make investigations and proceedings quicker and cheaper, but also, and what is even more important, more efficient. Speech content analysis is one of the most promising approaches to distinguish between lies and truths, and it already has a long history dating back to around 900 BC [4]. The underlying assumptions in verbal lie detection are that truth tellers exhibit coherence between statement and belief, whereas liars experience a discrepancy between the two[5], that liars have to think harder and that they try more than truth-tellers to make a convincing impression[4], and that people talk in different ways about events which are based upon their experience in comparison to what they only have imagined and fabricated [3,4,5].As a result of previous research there are several known speech content criteria—based techniques available today. However, previous research on statement assessment has focused on mainly one of them, namely the Statement Validity Assessment (SVA) method. Although the SVA method was originally designed to determine the credibility of child witnesses' testimonies in trials for sexual offences it is today the most extensively used worldwide for evaluating the veracity of also adults’ testimonies. Also, more than 50 empirical studies about this method have been published up to date, considering mainly adult witnesses' and plaintiffs' accounts [2,5,6,7,8,9]. Multivariable Adults' Statements Assessment Model (MASAM) was more recently designed as a tool for judging the credibility of adult witnesses' statements. Results of previous studies suggest, that MASAM is a useful tool discerning between memories of self-experienced real—life events and fabricated or fictitious accounts [10].

Previous studies on methods, using verbal content criteria have mainly focused on adult witnesses’ and plaintiffs’ accounts. Only very few studies have explored the possibility of using statement analysis techniques to detect false confessions and denials [11]. Moreover, no reliable data regarding the accuracy of SVA assessments in real-life cases are currently available [4]. The aim of this study was therefore to examine if SVA and/or MASAM can be used in differentiating suspects’ true and false accounts.

### Statement Validity Assessment and Multivariable Adults' Statements Assessment Model

SVA is a comprehensive procedure for generating and testing hypotheses about the source of a given statement in nine diagnostic steps [12]. The core of the technique is assessment of the presence of 19 CBCA criteria in the transcribed interview [2,3,5,6,7,8]. Each criteria is assumed to occur more frequently in truthful than deceptive accounts. According to the theory on which the selection of the 19 CBCA criteria is based, such criteria are likely to indicate genuine experiences because they are typically too difficult to fabricate [5,12]. Validity Checklist has been developed to explore variables other than truthfulness that may affect veracity of the statement and to consider alternative interpretations of the CBCA outcomes [2,5,12,13].

More than 50 empirical studies and few meta—analysis on SVA have been published to date, mainly with adult participants [2,3,4,5,6,7,8,14,15]. Those studies demonstrate that SVA analyses can be useful for lie detection purposes, since truth-tellers generally obtain significantly higher total scores than liars [16,17,18,19,20] and positive effect sizes have been observed in each and all of the credibility criteria [7,14]. Thus, the studies show that SVA is significantly more accurate than veracity assessment without using specialized tools [2,4,15] and evaluators using the SVA criteria achieve higher hit rates than clinical psychologists assessing reliability of the accounts of physical and psychological symptoms made by clients in legal settings [9]. However, in one of the studies adult truth-tellers obtained lower CBCA scores than liars [4,20]. In the, still very few, studies on suspects' accounts, only some individual CBCA criteria showed differences, that is: self—depreciations and doubts about own testimony were more often present in deceptive accounts, and unexpected complications were more often present in true accounts [11]. Caso et al. [16] found that lies included more description of conversations and subjective mental states, and Lee, Klaver and Hart [21] found that contrary to the SVA assumptions spontaneous corrections occur more often in lies than in truths.

The theoretical framework of MASAM is the same as of SVA, but hypotheses underlying content analysis according to MASAM are complemented with four additional assumptions in comparison to the SVA [10]. First, it is necessary to consider the fact that in each statement, truthful and deceptive, true and false information can be found. Moreover, if a witness’ intention is to give untruthful picture of actual events, certain differences appear in form and content of the given statement; the truthful parts will differ from lies. Furthermore, it is necessary to refer to the course of statement formation, that is to analyze circumstances of the event (what happened?), to establish witness’ characteristics (who gave the statement?) and to assess the course of the interview (how was the witness interviewed?). In accordance to MASAM assumptions high evidential value of a statement is based on and proven (supported?) by coherence between (1) the content and form of a statement, (2) the object and event features (i.e. complexity and observation conditions), (3) observer's characteristics (i.e. witness' prior experiences, age) and (4) interviewing circumstances (i.e. how detailed was the interview, when was the witness interviewed). It should specifically be mentioned, that, contrary to the CBCA technique, MASAM does not assume that in witness's statement which describes one's experiences, certain elements should be found [22].

There are 21 MASAM content criteria, divided into three categories: general features, details and deposition. Each of the content criteria is assessed separately with respect to object and event features (what happened?), observer's characteristics (who is the suspect?) and interviewing circumstances (how was the witness questioned?). Verbal cues are supposed to assist analysis of respective areas related to formation of statements as well as to be a kind of a guide to direct attention to areas that require more detailed analysis.

Each verbal cue of MASAM is analysed with reference to: what happened, who is the suspect and how was the witness or suspect interviewed? A decision algorithms provides raters with accurate guidelines regarding the way in which the results of content analysis conducted with the use of MASAM criteria should be interpreted [10]. The algorithm has been developed to enhance the accuracy of the method, since the first research, conducted in 2012 [22,23], showed, that MASAM distinguishes adults' truthful statements from lies on the average level of accuracy of 69.22% (72.00% for truthful statements and 54.17% of false statements) when raters only use their own judgment. However, as the MASAM decision algorithms were developed in 2015, the level of accuracy reached almost 99.95% of experience—based statements and 90% of invented statements [10]. Until now, MASAM has not yet been tested on suspects' accounts. SVA and MASAM content criteria are presented in Table 1.

**Table 1:** Statement Validity Assessment (SVA) [2,12] (and Multivariable Adults' Statements' Assessment Model (MASAM)[10,22].

SVA criteria			MASAM criteria		
	Name	Description		Name	Description
Criteria—Based Content Analysis					
1	Logical structure	statement contains no contradictions or logical inconsistencies	1	Internal coherency	internal structure of a statement, appearance or lack of contradictory remarks or self-contradictory elements
2	Unstructured production	suspect reports various elements of an incident in an unsystematic, chronologically disorganized manner	2	Coherence with other statements	appearance or lack of contradictions to other depositions given by other suspects during proceeding
3	Quantity of details	the event, the location and surroundings, and the people involved are described in great detail	3	Coherence with other evidence	appearance of lack of contradictions to information established on the basis of other evidence
4	Contextual embedding	the event in question is described as related to particular locations, time schedules, personal relationships	4	Volume of statement	amount of information, data, details, descriptions provided by witness and assessment of how vast is deposition
5	Descriptions of interactions	events are characterised by a sequence of actions and reactions	5	Description language	features of language used by suspect, level of linguistic capability, accuracy, uniqueness of description and phrasing
6	Reproduction of conversation	suspect reproduces conversations between different persons, using particular speech behaviour, vocabulary, etc. of those persons	6	Structure of statement	hierarchical, cause–effect, chronological
7	Unexpected complications during the incident	the course of events is interrupted by unexpected complications and obstacles	7	Statement linguistic function	descriptive, expressive, persuasive, contact building, meta textual;
8	Unusual details	refers to particular elements or details of a statement which are unexpected, surprising, e.g. odd details	8	Character and types of details	appearance or lack of irrelevant details unexpected, extraordinary, description of unexpected complications
9	Superfluous details	details which are not strictly necessary for the description of the incident	9	Interactions descriptions	appearance and frequency of recapturing cause–effect chains
10	Accurately reported details misunderstood	details and actions are reported which are obviously not understood by the suspect, in particular their meaning and function	10	Consequences	appearance of information on alleged and factual consequences of described events; suspect's awareness, adequacy of suspect's assessments
11	Related external associations	suspect describes events which refer to earlier events which are in some way related to the incriminated actions	11	Contextual setting and external associations	appearance and adequacy of information on circumstances in which event took place
12	Accounts of subjective mental state	the descriptions of suspect's feelings and cognitions, their development and changes during the event	12	Sensory data	presence and adequacy of proportion of sensory data (visual–auditorial–smell–taste–sensational)
13	Attribution of victim's mental state	descriptions of emotions, cognitions and motivations which were attributed by the suspect to the victim	13	Source of statement	memory statement is based on data originating from one or several receptors, senses
14	Spontaneous corrections	suspect corrects or modifies previous descriptions without having been prompted by the interviewer	14	Description of internal states	appearance of emotions and/or thoughts, character of thoughts and/or emotions, their level of intensity
15	Admitting lack of memory	suspect expresses concern that he or she may not remember all relevant details, that the description of particular details may be incorrect	15	Descriptions of relations	suspect describes and explains or avoids and skips one's relation to events, to people involved in event, to the course of event and its causes
16	Raising doubts about one's own statement	suspect indicates that part of his or her descriptions sounds odd, implausible, unlikely, he or she can hardly believe that this is a correct account of what had happened	16	Readiness to depose	suspect's attitude towards hearing is characterized with readiness to describe, recollect, rehearse main plots as well as by-plots
17	Self—depreciation	suspect mentions personally unfavourable, self—incriminating details	17	Readiness to search, identify, and reproduce memory traces	suspect's willingness to search in one's memory in order to find information necessary to answer questions
18	Pardoning the victim	suspect excuses the victim for his or her behaviour	18	Level of confidence	internal doubts about memories
19	Details specific to a type of crime	suspect describes elements which are typical for this type of crime but, on the other hand, are counter intuitive for the general public or discrepant to everyday knowledge or stereotypes	19	Complementing	readiness to complement statement through answering detailed questions or constant refusal to make answers precise or to search for and relate on additional aspects of an event
Validity Checklist				20	Memory loses	appearance and character of memory deficits; areas of description that are blurred, suspect's awareness
20	Inappropriateness of language and knowledge	suspect's use of language and display or knowledge beyond the normal capacity of a person his or her age or beyond the scope of what the suspect may have learned from the incident	21	Search for acceptance	appearance or lack of phrasing which reveal suspect's need of being accepted or understood by interviewer
21	Inappropriateness of affect	refers to whether the affect displayed by the suspect when being interviewed is inappropriate for the suspect's experiences			
22	Susceptibility to suggestion	refers to whether suspect demonstrates any susceptibility to suggestion during the interview			
23	Suggestive, leading or coercive questioning	examines whether the interviewer put suggestions to the interviewee or exerted any kind of pressure			
24	Overall inadequacy of the interview	other factors determining quality of the interview			
25	Questionable motives to report	refers to whether the suspect may have questionable motives to report the incident in certain way			
26	Questionable context of the original disclosure or report	refers to the origin and history of the statement, particularly the context of the first report			
27	Pressures to report falsely	deals with the question of whether there are indications that others suggested, coached, pressured or coerced the suspect to make a false report			
28	Inconsistency with the laws of nature	refers to describing events that are unrealistic or impossible			
29	Inconsistency with other interviewee's descriptions	refers to the possibility that major elements in the description of the core of the event are inconsistent with or contradicted by another statement made by the suspect or somebody else			
30	Inconsistency with other evidence	refers to the possibility that major elements in the statement are contradicted by reliable physical evidence or other concrete evidence			

*Note*: each of the MASAM criteria is assessed separately with respect to object and event features (what happened?), observer's characteristics (who is the suspect?) and interviewing circumstances (how was the witness questioned?).

## The experiments

The overall purpose of the experiments was to examine whether and to what extent SVA and MASAM can be used to improve accuracy in assessments of the veracity of suspects statement. In line with previous findings regarding both MASAM and SVA’s potential in distinguishing between true and false witness statements it was hypothesized that:

*Hypothesis 1*) Veracity assessments using MASAM and SVA are significantly more accurate than veracity assessments that are not based on any of the techniques.

*Hypothesis 2)* True suspect statements will receive significantly higher overall scores than false suspect statements, using both MASAM and SVA.

## Experiment 1—Materials and methods

### Participants

Study participants were in total 39 law students (21 women, 18 men) from Uppsala University, Sweden. The participants were taking a course in Law and Psychology in which statement analysis was part of the curriculum. Decision as to whether or not to take part in this study was completely voluntary. Subjects were informed, that the aim of the research is to study content analysis methods. Participants received no compensation or remuneration for taking part in the research. Participants’ ages varied between 23 and 27 years (M = 24.80 years, SD = 1.27). Two thirds of the participants (*n* = 26) received training in MASAM whereas the other third did not (*n* = 13). There were no signicicant group differences between trained and untrained raters for age, gender, education or other sample characteristics.

### Training

The participants who did receive training took part in a three day course, in total 20 hours, regarding statement analysis. The course included general themes such as definitions of truths and lies, theoretical assumptions of content analysis techniques and practical exercises using the MASAM method for statement analysis. The trainer explained and illustrated each MASAM criterion in depth and also discussed them with the students. Students were encouraged to ask questions and take active part in the training. Completion of the training was a precondition for conducting the analysis of the suspects’ statements included in the study.

The participants who did not receive any training were encouraged to assess the statements using their own judgements. They did not listen to any lecture, neither regarding lie detection in general nor about specific methods for statement analysis.

### Design

The experiment had a 2 (*analysis method*: MASAM vs. own judgements) x 2 (*statement veracity*: true vs. false) x 2 (*suspect attitude*: confession v. denial) mixed design. Whereas the *analysis method* variable was a between subjects factor, the variables *statement veracity* and *suspect attitude* were within subject factors. However, since real suspect statements were used, their veracity could not be manipulated. We discuss the implications of this in Limitations.

### Material

The material used for statement analysis consisted of 89 transcripts of suspect interrogations recorded in real life criminal cases. The transcripts were obtained from District Courts as well as Appelate Courts that were asked to provide criminal case files. The case files were carefully reviewed to select appropriate cases in the four following categories: 1) True denials (N = 17), 2) False denials (N = 173), 3) True confessions (N = 170) and 4) False confessions (N = 8). Known cases of for instance false confessions or any of the other categories could not be used since the participants’ answers most probably would have been effected by their knowledge of the outcome in the real case. Instead the criteria for inclusion was, in cases of true statements, that there was overwhelming evidence independent of the suspect’s statement which corroborated it and, in cases of false statements, that there was overwhelming evidence independent of the suspect’s statement which refuted it. This criteria for inclusion seemed reasonable provided that on the one hand, ground truths are usually unobtainable in criminal cases but on the other hand, making correct classifications of the transcripts was crucial for the study’s validity. Factors such as the type of crime and the suspect’s gender, age and ethnicity varied across the transcripts.

### Procedure

All participants received 8 interrogation transcripts to assess. They were instructed to read the transcripts carefully and to not communicate with one another. The deadline for submitting their analyses was one week later. The participants who used MASAM for analysis rated all statements with reference to the individual MASAM-criteria, from which an overall score was calculated. By the end of their assessment, participants also indicated whether they thought the suspect statement was true or false. The participants who did not use MASAM were simply asked to indicate whether they thought the suspect statement was true or false.

## Results

### Judgment accuracy rates

Analysis of accuracy rates for identifying truthful and untruthful accounts indicated that training significantly affected accuracy. Untrained subjects classified correctly 67of 113 of suspects' accounts (59.29%), including 31 of 58 true statements (53.45%) and 36 of 55 false statements (65.45%). Coders from the Uppsala University using MASAM reached a total accuracy rate of 70.03% (250 out of 357), truthful accounts were correctly classified in 77.53% of the cases (144 among 183) and false statements were correctly classified in 61.22% of the cases (106 among 174). Veracity assessment with the use of MASAM was significantly more accurate than classification without training (*χ*^*2*^ = 5.98, p < .02), but only for truthful statements (*χ*^*2*^ = 12.54, p < .01) and not for the false suspects' statements (*χ*^*2*^ = .317, p = .57).

### Differences in MASAM scores between true and false statements

Coders were asked to rate 63 MASAM criteria on 6-point scale (if a criterion was not present in the statement it received a score of 1, and if it was strongly present, it received score of 6), therefore MASAM scores could range from 63 to 378. According to the MASAM assumptions, the higher the overall score, the higher the probability that the statement is experience based and not fabricated. To test our predictions that MASAM overall results will be higher for truthful suspects' accounts we conducted a two (veracity: truthful, false) x two (suspect's attitude: denial, confession) mixed ANOVA. The veracity x suspects' attitude interaction was insignificant (F(1,367) = .334, p = .56) and there was no main effect of suspect's attitude on overall result (M_confession_ = 306.60, SD_confession_ = 48.73; M_denial_ = 283.23, SD_denial_ = 58.15; F(1,367) = .051, p = .82). However, there was main effect of statements' veracity on overall result (M_true_ = 308.49, SD_true_ = 48.19; M_false_ = 280.16, SD_false_ = 57.91; F(1,367) = 6.63, p < .02). A *post hoc* Tukey's HSD test showed that false denials differed significantly from true denials (p < .04) and true confessions (p < .001), but false confessions were not rated significantly different from false denials (p = .99), true denials (p = .47) and true confessions (p = .61).

### MASAM individual criteria

To assess the effectiveness of the individual MASAM criteria and test the significance of differences between truthful and false accounts the one-way ANOVA across individual criteria were carried out. Analysis of variance procedure gave statistically significant results for MASAM, *F* = 2.56, *p* < .001, *η2* = .21. This significant effect of veracity indicates that for at least some individual criteria difference between truths and lies emerged, however chosen predictor explains only small part of the variance. To further explore differences between experience—based and fabricated suspects' accounts we have tested significance of differences for individual MASAM criteria, which are presented in Table 2.

**Table 2:** MASAM scores as function of veracity—law students.

MASAM content criteria	Level of analysis	True (n = 189)		False (n = 183)		*F*	Cohen's *d*
		*M*	*SD*	*M*	*SD*		
Internal coherency	objects/events	4.78	1.78	4.40	1.82	7.21**	.21
	suspect	5.24	1.41	4.74	1.65	19.09**	.33
	interview	5.23	1.48	4.95	1.53	5.92*	.19
Coherence with other statements	objects/events	5.61	1.18	5.31	1.46	9.77**	.23
	suspect	5.66	1.10	5.36	1.38	11.63**	.25
	interview	5.68	1.08	5.43	1.30	8.59**	.22
Coherence with other evidence	objects/events	5.54	1.23	4.80	1.84	41.97**	.48
	suspect	5.57	1.18	4.91	1.74	37.85**	.46
	interview	5.60	1.12	5.02	1.69	32.15**	.42
Volume of statement	objects/events	4.54	1.69	3.90	1.61	22.05**	.38
	suspect	5.05	1.40	4.41	1.55	29.49**	.43
	interview	5.41	1.19	4.84	1.47	29.01**	.43
Description language	objects/events	5.26	1.15	4.86	1.43	16.07**	.32
	suspect	5.28	1.21	4.89	1.47	13.35**	.29
	interview	5.67	0.81	5.36	1.24	13.67**	.30
Structure of statement	objects/events	5.17	1.34	4.84	1.49	8.88**	.24
	suspect	5.44	1.04	5.17	1.27	8.49**	.24
	interview	5.69	2.41	5.32	1.30	4.44*	.18
Statement linguistic function	objects/events	5.19	1.36	4.72	1.49	17.09**	.33
	suspect	5.39	1.11	4.86	1.43	29.58**	.42
	interview	5.48	1.18	5.16	1.35	9.53**	.26
Character and types of details	objects/events	4.35	1.79	3.90	1.76	9.25**	.25
	suspect	4.94	1.47	4.29	1.65	26.62**	.41
	interview	5.19	1.42	4.82	1.52	8.56**	.25
Interactions	objects/events	4.19	1.85	3.59	1.74	17.25**	.32
	suspect	4.83	1.54	4.18	1.66	26.23**	.41
	interview	5.28	1.31	4.94	1.46	8.44**	.24
Consequences	objects/events	4.22	1.83	3.88	1.74	4.56*	.19
	suspect	4.66	1.65	4.24	1.65	8.67*	.25
	interview	5.29	1.27	4.99	1.45	6.47*	22
Contextual setting and external associations	objects/events	4.47	1.75	3.85	1.67	21.25**	.36
	suspect	4.91	1.51	4.30	1.52	25.75**	.40
	interview	5.26	1.35	4.89	1.39	12.29**	.27
Sensory data	objects/events	4.62	1.68	4.04	1.80	19.24**	.33
	suspect	4.96	1.48	4.31	1.73	26.79**	.40
	interview	5.39	1.22	4.97	1.54	15.21**	.30
Source of statement	objects/events	4.89	1.64	4.44	1.80	12.68**	.26
	suspect	5.23	1.36	4.73	1.61	20.32**	.33
	interview	5.54	3.05	5.20	1.39	2.73	.13
Description of internal states (emotions and/or thoughts)	objects/events	4.07	1.78	3.41	1.71	21.97**	.37
	suspect	4.55	1.67	3.81	1.74	31.55**	.43
	interview	5.22	1.33	4.91	1.49	7.54**	.22
Descriptions of relations	objects/events	4.73	1.60	3.96	1.74	34.92**	.46
	suspect	5.12	1.33	4.46	1.57	34.13**	.45
	interview	5.38	1.20	4.96	1.46	15.92**	.32
Readiness to depose	objects/events	4.98	1.51	4.11	1.74	47.24**	.53
	suspect	5.33	1.19	4.41	1.67	69.69**	.63
	interview	5.45	1.09	4.83	1.52	37.61**	.48
Readiness to search, identify, and reproduce memory traces	objects/events	4.82	1.56	3.89	1.79	54.08**	.55
	suspect	5.16	1.32	4.31	1.68	57.80**	.56
	interview	5.45	1.14	4.98	4.13	4.64*	.18
Level of confidence—internal doubts about memories	objects/events	4.88	1.53	4.42	1.59	14.96**	.29
	suspect	5.22	1.28	4.74	1.45	22.48**	.35
	interview	5.45	1.10	5.00	1.36	23.01**	.37
Complement	objects/events	4.88	1.60	4.33	1.78	17.41**	.33
	suspect	5.21	1.32	4.56	1.68	31.19**	.44
	interview	5.49	1.07	4.88	1.60	33.34**	.46
Memory loses	objects/events	4.78	1.66	4.38	1.71	10.59**	.24
	suspect	5.25	1.28	5.00	4.05	22.17**	.09
	interview	5.33	1.29	5.10	2.26	3.07	.13
Search for acceptance	objects/events	4.68	1.82	4.44	1.73	3.44	.14
	suspect	4.97	1.63	4.65	1.68	6.75**	.19
	interview	5.26	1.41	5.16	1.35	0.86	.07

Note

* p < .05

** p < .01.

As can be seen in Table 2 there were significant differences between truths and lies assessed by the University of Uppsala law students for 58 out of 63 MASAM criteria. Effects were small for most of the individual criteria, only for *readiness to depose* rated with reference to *objects and events* (*p* < .01, *d* = .53) and *suspect* (*p* < .01, *d* = .63), as well as *readiness to search*, *identify and reproduce memory traces* rated with reference to *objects and events* (*p* < .01, *d* = .55) and *suspect's characteristics* (*p* < .01, *d* = .56) medium effects were observed.

## Experiment 2—Materials and methods

### Participants

Study participants were in total 34 psychology students (30 women, 4 men) from University of Silesia in Katowice, Poland. The participants were taking a course in forensic psychology in which statement analysis was part of the curriculum. Participants’ ages varied between 21 and 33 years (M = 22.92 years, SD = 2.13).For each participant decision as to whether or not to take part in this study was completely voluntary. Subjects were informed, that the aim of the research is to study validity and accuracy of content analysis methods. Participants received a consent form (for participation to the study) prior to the study and a debriefing form after the end of the study. Participants received no compensation, remuneration or credit for taking part in the research. At the beginning of the study participants declared number of content analysis they are ready to conduct and were randomly assigned to one of the groups. There were no significant sample characteristics differences between trained and untrained group. Proportions between the groups of raters were set to assure that similar number of ratings from untrained raters, participants using MASAM and subjects using SVA could be collected during the study.

### Training

Most of the participants (*N* = 24) received training in SVA and MASAM, the rest assessed veracity without previous training and not using any of the content analysis technique (*N* = 10). The participants who did receive training took part in a fourday course, in total 26 hours, regarding statement analysis. The course included general themes such as definitions of truths and lies, theoretical assumptions of content analysis techniques and practical exercises using the SVA and the MASAM method for statement analysis. The trainer explained and illustrated each of the SVA and MASAM criterion in depth and also discussed them with the students. Students were encouraged to ask questions and take active part in the training. Completion of the training was a precondition for conducting the analysis of the suspects’ statements included in the study.

### Design

The experiment had a 3 (*analysis method*: SVA vs. MASAM vs. own judgements) x 2 (*statement veracity*: true vs. false) x 2 (*suspect attitude*: confession v. denial) mixed design. Whereas the *analysis method* variable was a between subjects factor, the variables *statement veracity* and *suspect attitude* were within subject factors. However, since real suspect statements were used, their veracity could not be manipulated. We discuss the implications of this in Limitations.

### Material

Data were transcripts of suspects interrogations conducted in 104 Polish criminal proceedings, made available by the District and Regional Courts of the Silesian voivodeship in response to the researchers' request. Individual files were chosen by courts' authorities and secretarial staff, researchers did not have influence on their quantity and quality. From the court cases only the ones which ended in judgment in force and statements given during the proceedings were evaluated by both instances courts and that evaluations were foundations to conduct factual adjudication of case. Information on evidential material which was collected during the proceedings, including; demonstrative evidence, photographs, medical certificates, experts' opinions, as well as significance of each evidence in terms of courts' ruling and coherence between them and offender's statement content was included in taxonomic sheet. Statements which were recorded in protocols were transcribed and raters received them in the anonymized form.

The offenses discussed in the interrogations varied from theft to homicide. Suspects whose statements were subjected to content analysis were in 43.97% women, 56.03% men and aged from 17 to 74 years (M = 36, SD = 15). The content of statement given by a suspect throughout whole proceeding (during from 1 to 7 interrogations, M = 2.51, SD = 1.19). Truthful statements' volume was smaller than untruthful ones, a statistically significant differences was observed between volume (*F*(1,193) = 50.65, *p* < .001, *ƞ*^*2*^ = .0864).Psychology students from the University of Silesia rated confessions with the use of SVA in 141 cases (40.40%) and denials in 208 cases (59.60%), 262 statements were true (75.07%), suspects were apprehended in 64.47% cases (N = 225). For MASAM ratings 138 confessions (41.32%) and 196 denials (58.68%) were used, of which 257 were true (76.94%) and 77 were false (23.06%); suspects were apprehended in 219 cases (65.57%). Untrained participants analyzed 126 confessions (42%) and 174 denials (58%), 228 assessed statements were true (76%) and 72 were false; suspects were apprehended in 102 cases (34%).Since we have gained access to the files chosen and made available by the courts, it was not possible to keep the balance between four categories of statements. As a result, true denials were analyzed 240 times (35.14%), false denials 164 times (24.01%) true confessions were analyzed 279 times (40.85%), and no false confessions were assessed in the study.

### Procedure

MASAM and SVA questionnaire used to evaluate statements included a table which presented content criteria of given method with a short characteristic (description of certain criteria meaning). A 6—point scale to evaluate level of coherence between statement and given criteria was used. In our study one indicated that the criterion was not at all present or fulfilled and 6 indicated that the criterion was present or fulfilled to a very high degree. Each of 21 MASAM criteria was rated with the use of six-point scale (in which “1” signifies absolute inconsistency while “6” signifies total coherence) in three areas: coherence with objects and events features, coherence with suspect's characteristics and coherence with interview circumstances. Content analysis of statements with the use of SVA and MASAM was conveyed in reference to all content criteria adequate to the technique. Evaluation included all 30 of SVA and 21 MASAM criteria.

Raters evaluated each criteria individually and added up points gained by each criteria. In final part of the worksheet there were columns which allowed to evaluate evidential value of a statement based on method (true/false). Alternatively, evaluator could assess veracity of the statement independently from content analysis results (true/false) and add some remarks.

## Results

### Judgment accuracy rates

Analysis of accuracy rates for identifying truthful and untruthful accounts indicated that both training and content analysis technique significantly affected accuracy. Untrained subjects classified correctly 89 of 300 of suspects' accounts (29.67%), including 74 of 228 true statements (32.45%) and 15 of 72 false statements (20.83%). SVA was accurate in 60.17% of the cases (210 of 349), but this technique was more accurate in detecting lies resulting in 68.96% of right classifications (60 of 87), whereas in detecting truths accuracy rate was 57.25% (150 of 262). The average accuracy of raters from the University of Silesia MASAM—trained subjects was 70.66% (236 of 334), for truths 75.48% (194 of 257) and for lies 54.55% (42 of 77). The overall accuracy of MASAM veracity assessment was significantly higher than both: rating with the use of SVA (*χ*^*2*^ = 8.28, p < .005) and judgment without training (*χ*^*2*^ = 106.12, p < .001); SVA ratings were significantly more accurate than ratings without the use of content analysis method (*χ*^*2*^ = 60.31, p < .001). Although veracity assessment of true statements with the use of MASAM was significantly more accurate than classification with the use of SVA (*χ*^*2*^ = 19.25, p < .001), there were no significant differences between MASAM and SVA when false statements were analyzed (*χ*^*2*^ = 3.585, p = .0583).

### Differences in SVA and MASAM scores between true and false statements

#### SVA and MASAM overall ratings

To test our predictions that SVA and MASAM overall results will be higher for truthful suspects' accounts we examined overall means. Coders were asked to rate 30 SVA criteria and 63 MASAM criteria on 6-point scale (if a criterion was not present in the statement it received a score of 1, and if it was strongly present, it received score of 6), therefore SVA scores could range from 30 to 180 and MASAM scores could range from 63 to 378. According to the SVA and MASAM assumptions, the higher the overall score, the higher the probability that the statement is experience based and not fabricated.

**Fig 1:**
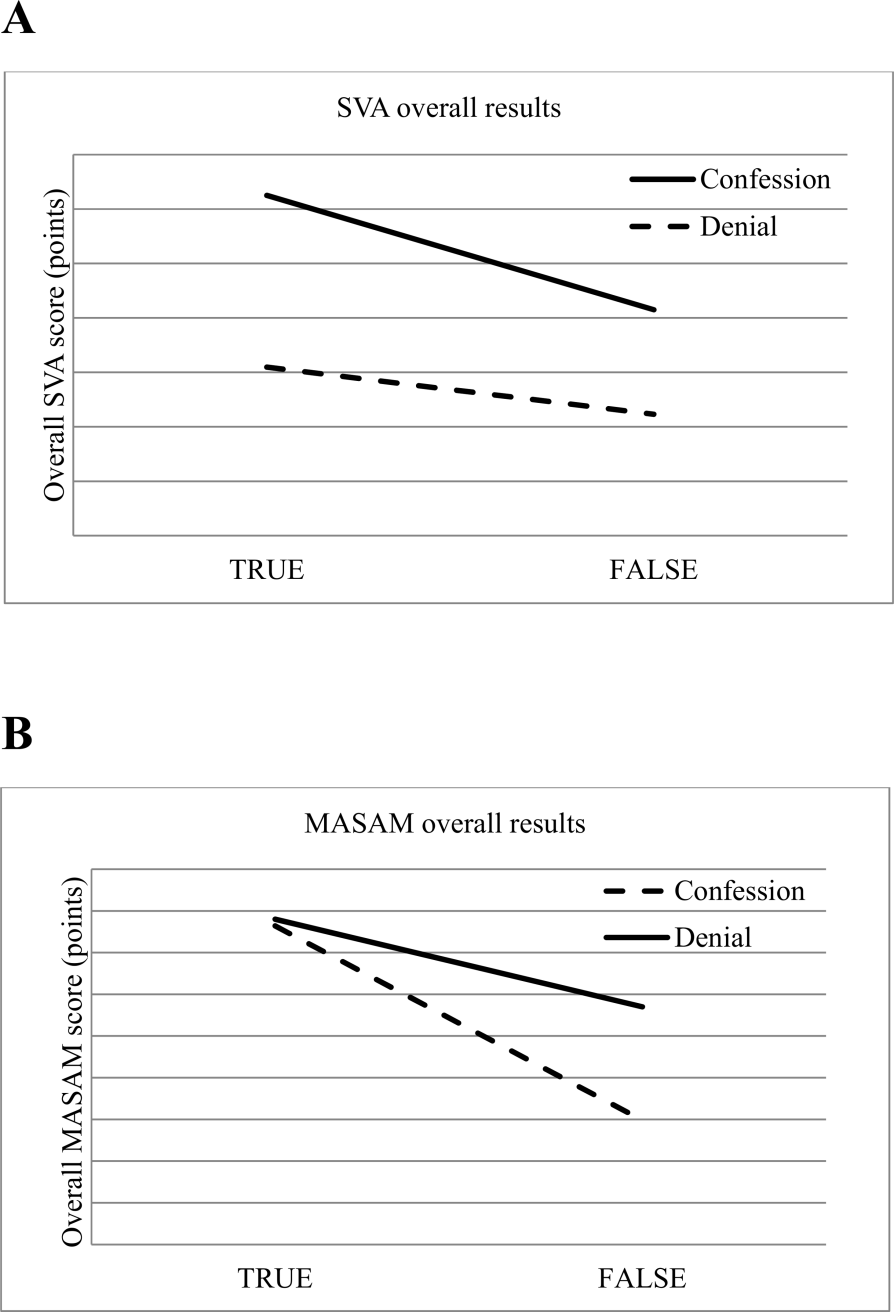
**Differences between true and false confessions and denials rated with SVA—overall results (Fig 1A.) and differences between true and false confessions and denials rated with MASAM—overall result (Fig 1B.);** Note: for SVA: *F*(1,347) = .67; *p* = 0,42; for MASAM: *F*(1,334) = 4,47; p < .05.

To test our predictions that MASAM and SVA overall results will be higher for truthful suspects' accounts we conducted a two (veracity: truthful, false) x two (method: denial, confession) and a two (suspect's attitude: confession, denial) mixed ANOVAs (Fig 1A and 1B). The veracity x content analysis interaction was insignificant (F(1,679) = 1.25, p = .26), but there was a main effect of method on overall result (SVA: M_true_ = 107.58, SD_true_ = 14.67; M_false_ = 102.46, SD_false_ = 15.25; MASAM: M_true_ = 332.08, SD_true_ = 40.20; M_false_ = 320.84, SD_false_ = 43.30; F(1,679) = 6543.33., p < .001). A *post hoc* Tukey's HSD test showed that true and false statements rated with the use of SVA did not differ significantly (p = .53), whereas overall results of MASAM analysis differed significantly for true and false statements (p < .001). The suspects' attitude x method interaction was insignificant (F(1,679) = 5.43, p = .02), but there was a main effect of overall result for both method (F(1,679) = 9198.30, p<0.001) and suspect's attitude (F(1,679) = 28.52, p<0.001). *Post hoc* comparisons using the Tukey's HSD test indicated that the mean score for confession and denials when assessed with the use of MASAM was significantly different (M_confessions_ = 340.01, SD_confessions_ = 31.09; M_denials_ = 322.09, SD_denials_ = 45.58, p < .001). However, confessions and denials did not significantly differ when assessed with the use of SVA (M_confessions_ = 110.50, SD_confessions_ = 14.12; M_denials_ = 103.46, SD_denials_ = 14.88, p = .14).

#### SVA and MASAM individual criteria

To assess the effectiveness of the individual SVA and MASAM criteria and test the significance of differences between truthful and false accounts the one-way ANOVA across individual criteria were carried out. Analysis of variance procedure gave statistically significant results for SVA (*F* = 3.06, *p* < .001, *η*^*2*^ = .23) and MASAM (*F* = 2.56, *p* < .001, *η*^*2*^ = .21). This significant effect of veracity indicates that for at least some individual criteria difference between truths and lies emerged, however chosen predictor explains only small part of the variance. To further explore differences between experience—based and fabricated suspects' accounts we have tested significance of differences for individual SVA and MASAM criteria, which are presented in Table 3.

**Table 3:** SVA scores as function of veracity—psychology students.

SVA content criteria	True (n = 262)		False (n = 87)		*F*	Cohen's *d*
	*M*	*SD*	*M*	*SD*		
Logical structure	5.01	1.65	4.34	1.93	9.79**	.38
Unstructured production	3.51	2.12	3.30	2.10	0.68	.11
Quantity of detail	3.82	1.96	3.49	1.99	1.76	.16
Contextual embedding	3.40	2.12	2.44	1.90	14.10**	.46
Descriptions of interactions	2.32	1.90	2.56	2.03	1.09	-.13
Reproduction of conversation	2.03	1.80	2.80	2.12	11.13**	-.41
Unexpected complications	2.47	2.05	2.56	2.04	0.12	-.04
Unusual details	1.69	1.48	1.84	1.62	0.63	-.09
Superfluous details	2.71	2.00	2.33	2.03	2.25	.18
Accurately reported details misunderstood	1.35	1.13	1.19	0.77	1.43	.15
Related external associations	1.40	1.19	1.37	1.18	0.06	.03
Accounts of subjective mental states	2.28	1.85	2.16	1.83	0.28	.07
Attrubution of victim's mental state	1.62	1.31	1.80	1.49	1.19	-.13
Spontaneous corrections	1.98	1.76	2.15	1.77	0.57	-.09
Admitting lack of memory	4.00	2.18	4.27	2.07	1.07	-.13
Raising doubts about one's own testimony	2.24	1.65	1.86	1.40	3.70	.24
Self—depreciation	3.54	1.97	2.18	1.52	34.35**	.69
Pardoning the victim	3.07	1.53	2.56	1.16	7.97**	.34
Details characteristic of the offense	5.12	1.50	4.62	1.68	6.73**	.32
Appropriateness of language and knowledge	5.71	0.76	5.48	1.22	4.18*	.25
Appropriateness of affect	4.69	1.37	4.67	1.37	0.02	.02
Interviewee's sucsceptibility to suggestion	4.46	1.39	4.42	1.44	0.04	.03
Evidence of suggestive questioning	4.89	1.33	4.75	1.40	0.72	.11
Overall adequacy of the interview	4.87	1.33	4.56	1.51	3.17	.22
Motives to report	5.42	1.11	5.33	1.24	0.34	.07
Context of the original disclosure	5,22	1.35	5.26	1.34	0.05	-.03
Pressure to report falsely	5,23	1.18	5.13	1.34	0.49	.09
Consistency with the laws of nature	5,44	1.22	5.09	1.55	4.72*	.27
Consistency with other statements	4,16	1.37	4.03	1.38	0.51	.09
Consistency with other evidence	3,93	1.32	3.86	1.41	0.15	.05

Note

* p < .05

** p < .01.

As can be seen in Table 3 only seven of the thirty SVA criteria were more often present in truthful accounts: *logical structure* (p < .01, *d* = .38), *contextual embedding*(p < .01, *d* = .46), *self depreciation*(p < .01, *d* = .69), *pardoning the victim* (p < .01, *d* = .34), *details characteristic of the offense* (p < .01, *d* = 0,32), *appropriateness of knowledge* (p < .05, *d* = .25) *and consistency with the laws of nature* (p < .05, *d* = .27); there were more *reproduction of conversation* in the false statements (p < .01, *d* = .41). However, magnitude of Cohen's *d* indicates only small and medium effects. Table 4 shows differences between truthful and untruthful statements rated with the use of MASAM by the psychology students of the University of Silesia.

**Table 4:** MASAM scores as function of veracity—psychology students.

MASAM content criteria	Level of analysis	True (n = 257)		False (n = 77)		*F*	Cohen's *d*
		*M*	*SD*	*M*	*SD*		
Internal coherency	objects/events	4.80	1.95	4.09	2.22	7.35**	.35
	suspect	5.35	1.45	4.92	1.83	4.53*	.27
	interview	5.26	1.61	4.99	1.77	1.59	.16
Coherence with other statements	objects/events	5.59	1.30	5.39	1.61	1.27	.15
	suspect	5.64	1.23	5.43	1.52	1.58	.16
	interview	5.64	1.23	5.52	1.41	0.56	.10
Coherence with other evidence	objects/events	5.61	1.22	5.39	1.53	1.71	.17
	suspect	5.64	1.17	5.53	1.33	0.52	.09
	interview	5.70	1.07	5.69	1.10	0.01	.02
Volume of statement	objects/events	4.81	1.76	4.30	1.92	4.79*	.28
	suspect	5.53	1.13	5.28	1.37	2.47	.21
	interview	5.75	0.76	5.34	1.27	12.13**	.44
Description language	objects/events	5.67	0.86	5.67	0.73	0.01	-.01
	suspect	5.66	0.94	5.75	0.84	0.54	-.09
	interview	5.86	0.55	5.83	0.59	0.15	.05
Structure of statement	objects/events	5.33	1.39	5.05	1.60	2.23	.19
	suspect	5.73	0.76	5.66	0.75	0.55	.10
	interview	5.68	0.99	5.45	1.32	2.59	.21
Statement linguistic function	objects/events	5.46	1.26	5.51	1.20	0.08	-.04
	suspect	5.77	0.62	5.74	0.75	0.13	.05
	interview	5.68	1.01	5.60	1.09	0.35	.08
Character and types of details	objects/events	4.40	1.98	4.16	2.05	0.92	.12
	suspect	5.23	1.43	5.08	1.62	0.66	.10
	interview	5.29	1.45	5.14	1.58	0.59	.10
Interactions	objects/events	4.19	2.04	3.79	2.10	2.23	.19
	suspect	5.14	1.57	5.02	1.63	0.36	.08
	interview	5.44	1.30	5.40	1.38	0.06	.03
Consequences	objects/events	4.10	2.10	3.89	2.17	0.65	.10
	suspect	4.68	1.85	4.60	1.82	0.13	.05
	interview	5.39	1.35	5.35	1.39	0.04	.03
Contextual setting and external associations	objects/events	4.77	1.82	4.21	2.00	5.40*	.30
	suspect	5.30	1.38	5.00	1.49	2.76	.22
	interview	5.45	1.24	5.26	1.37	1.29	.15
Sensory data	objects/events	5.04	1.62	5.13	1.68	0.17	-.05
	suspect	5.49	1.10	5.65	0.93	1.26	-.15
	interview	5.68	0.86	5.74	0.77	0.29	-.07
Source of statement	objects/events	4.94	1.73	4.82	1.89	0.27	.07
	suspect	5.47	1.17	5.52	1.06	0.11	-.04
	interview	5.49	1.28	5.48	1.23	0.01	.01
Description of internal states (emotions and/or thoughts)	objects/events	4.25	1.96	4.22	2.06	0.01	.01
	suspect	4.96	1.62	4.96	1.68	0,01	-.01
	interview	5.44	1.23	5.49	1.23	0.09	-.04
Descriptions of relations	objects/events	4.99	1.60	4.40	2.01	7.12**	.34
	suspect	5.53	1.01	5.39	1.29	0.99	.13
	interview	5.54	1.04	5.45	1.27	0.40	.08
Readiness to depose	objects/events	5.09	1.59	4.54	1.94	6.36*	.33
	suspect	5.56	1.01	5.22	1.52	5.22*	.29
	interview	5.69	0.85	5.42	1.31	4.75*	.28
Readiness to search, identify, and reproduce memory traces	objects/events	4.85	1.67	4.12	1.96	10.49**	.41
	suspect	5.32	1.26	5.17	1.23	0.89	.12
	interview	5.56	1.08	5.25	1.41	4.28*	.27
Level of confidence—internal doubts about memories	objects/events	4.79	1.71	4.35	1.88	3.73	.25
	suspect	5.32	1.32	5.27	1.18	0.08	.04
	interview	5.51	1.08	5.27	1.35	2.60	.21
Complement	objects/events	4.82	1.78	4.77	1.78	0.06	.03
	suspect	5.31	1.35	5.32	1.24	0.01	-.01
	interview	5.64	0.95	5.36	1.35	4.04*	.26
Memory loses	objects/events	4.66	1.86	4.32	1.94	1.86	.18
	suspect	5.43	1.22	5.35	1.25	0.23	.06
	interview	5.40	1.33	5.21	1.52	1.12	.14
Search for acceptance	objects/events	4.52	2.09	4.44	2.11	0.09	.04
	suspect	4.94	1.83	4.81	1.99	0.31	.07
	interview	5.25	1.55	5.36	1.41	0.34	-.07

Note

* p < .05

** p < .01.

Significant but small effects of veracity were found for eleven criteria: *internal coherence* rated with reference to *objects and events* (p < .01, *d* = .35) and *suspect* (p < .05, *d* = .27), *volume of statement* rated with reference to *objects and events* (p < .05, *d* = .28) and *interview* (p < .01, *d* = .44), *contextual setting and external associations* rated with reference to *objects and events* (p < .05, *d* = .30), *descriptions of relations* rated with reference to *objects and events* (p < .01, *d* = .34), *readiness to depose* (p < .05, *d* = .33/.29/.28), *readiness to search*, *identify and reproduce memory traces* rated with reference to *objects and events* (p < .01, *d* = .41) and *interview* (p < .01, *d* = .35) *and complement rated* with reference to *interview* (p < .05, *d* = .26).

## Discussion

The main purpose of the present study was to examine if SVA and/or MASAM could be useful in discriminating offenders' true and false statements. With only few exceptions, previous research has not explored the applicability of these techniques on statements by offenders [11]. In most of the previous studies incomplete SVA procedure was used [5] and MASAM validity has not been tested on statements given by suspects [10].

It has been shown, that the total accuracy rates obtained using SVA and MASAM are far higher than those obtained without using a specific assessment system. Trained coders are better at distinguishing between truths and lies than lay evaluators. It is perhaps surprising that after same training law students were more successful than psychology students in distinguishing between truthful and false statements. Our unpredicted result can be explained by differences in how interviews with suspects are recorded—in the Polish protocols only summary of suspect's answers are saved, whereas in the Swedish procedure notation is more detailed and reflects accurately suspect's answers. However, the approximately 70% accuracy rates obtained on the basis of SVA and MASAM ratings, although in line with previous research [2,3] do not suffice for an individual assessment.

The average total accuracy in our study is similar to results reported in research on witnesses' statements and suspects' accounts [2,3,24,25,26,27,28]. The differences in accuracy rates between lie detection without content analysis and SVA/MASAM coders support the idea that trained coders are better at distinguishing between truths and lies than lay evaluators. Results also show, that SVA is a better lie detection tool, than MASAM.

The basic assumption of SVA and MASAM is that a testimony derived from memory of an actual experience differs in content and quality from statement based on fabrication. It was hypothesized that there would be higher SVA and MASAM scores in truthful statements than in false. The overall pattern of results provides support for the validity of the SVA and MASAM techniques for detecting truthfulness of statements. In line with the Undeutsch hypothesis, the results showed a significant difference between total scores of truthful and false statements and it can be concluded that both veracity assessment techniques are useful in assessing veracity.

We found that only few of SVA criteria did differentiate between true and false explanations. Seven SVA criteria distinguished truthful and false statements and functioned as we expected: logical structure, contextual embedding, self—depreciation, pardoning the victim, details characteristic of the offense, appropriateness of language and knowledge and consistency with the laws of nature; we have also found more reproduction of conversation in lies than truths. These findings are somewhat in line with previous research [6,7,8]. However, conducting a SVA calls for more information than just the statement. It is assumed, that credibility assessment should be a result of an comprehensive ideographic approach and psychological experts using this technique should have access to all files containing the results of investigations by the police, the prosecutor and the court [5]. Accuracy and reliability of SVA could be also improved by taking into account case—specific conditions [5].

Vast majority of MASAM criteria differentiated between true and false explanations provided by the Swedish suspects with medium and small effect sizes. Only memory losses rated with reference to suspect's characteristic and source of statement evaluated with respect to interview among 63 MASAM criteria failed to discriminate between the memory of real self—experienced events and false or invented accounts. When statements from the Polish case files were analyzed only 10 of MASAM criteria discriminated significantly with medium or small effect sizes. Research has demonstrated that MASAM scores are related to external factors, such as interview style and method of offender's statement recording. Both SVA and MASAM are usually used to assess deception using detailed transcript or video of an interview, and this may therefore explain their apparent limitations when used in the manner adopted here.

### Limitations

Establishment of ground truth is essential when carrying out deception research [2]. In the present study the ground truth was not objectively established. Although there was a large sample size in this study and court records of each case were thoroughly analyzed to acknowledge that suspect's statement was false or truthful, we cannot rule out that suspects did not tell the truth as it happened and even when confessing had given a sweetened version of the crime.

In addition, previous research showed that discriminating efficacy was higher in field studies on sexual offences and intimate partner violence [6] and situational variables such as complexity of the event, time interval between event in question and interview and interview technique may affect veracity assessment outcome [5]. Therefore, there is a need for research focused on differences between true and false accounts made by offenders describing various crimes categories.

## Implications and future directions

Taking into consideration the established error rate of approximately 30% and the low effectiveness of the individual criteria as discriminators between self-experienced and invented memories, SVA and MASAM evaluators are not able to present the accuracy of their assessments as being beyond reasonable doubt, which is the standard of proof in criminal courts. Therefore, SVA and MASAM assessments of suspects' accounts are not accurate enough to be presented as scientific evidence in criminal courts.

We found that there were differences in quality and quantity of some individual criteria that distinguished truthful and deceptive statements. Further research can provide more evidence as to the apparent usefulness of the individual SVA and MASAM criteria with respect to individual characteristics of both true and fabricated statements [2].Further exploration of conditions under which low quality is to be found in truthful statements could enhance the possibility of more accurate differentiation between offenders' true and false accounts through a scientifically based technique for statement analysis. Specifically, it seems necessary to further explore and to estimate the impact of personal and situational variables on content quality. Moreover, previous studies indicate that there are qualitative and quantitative differences between true and false confessions [29].Future research is called to explore further differences between true vs false confessions and denials.

In order to standardise content analysis it is necessary to establish rules for using particular tools for analysing verbal cues and evaluators should be provided with clear guidelines concerning result interpretation. Authors of psychological content analysis techniques have not yet been able to develop reliable rules which would allow to determine when a content analysis should result in recognising a statement as experience-based and when it should be classified as false, based on invention or fantasy.
